# Colloids to improve diuresis in critically ill patients: a systematic review

**DOI:** 10.1186/2052-0492-2-37

**Published:** 2014-06-10

**Authors:** Simon JW Oczkowski, Ian Mazzetti

**Affiliations:** Division of Critical Care Medicine, McMaster University, Hamilton, ON L8N 3Z5 Canada; Critical Care Medicine Residency Program, Room 2U c/o Anesthesia Department, McMaster University, 1200 Main St. W., Hamilton, ON L8N 3Z5 Canada

**Keywords:** Albumin, Colloids, Critical care, Critical illness, Diuretics

## Abstract

**Background:**

The background of this study is to determine whether the addition of intravenous colloid to diuretic therapy, in comparison to diuretic therapy alone, improves diuresis and oxygenation and prevents intravascular volume depletion in intensive care unit (ICU) patients without shock.

**Methods:**

We searched MEDLINE, Embase, Cochrane Register of Controlled Trials, Google Scholar, conference abstracts of ACCP, SCCM, ATS, and references of relevant articles. Randomized controlled trials (RCTs) of adult ICU patients, not in shock (defined as patients on low dose or no vasopressors, without need for IV fluid bolus or blood transfusion within 24 h), comparing intravenous colloid therapy (human albumin, plasma, synthetic starches, or gels) plus diuretic to control (diuretic alone, or diuretic plus placebo). Two reviewers independently applied eligibility criteria, assessed quality, and extracted data.

**Results:**

Seven hundred fifty five studies were found in the initial search; 14 were deemed relevant; 2 were found to be eligible. There was good agreement between reviewers for study relevance (*k* = 0.869) and eligibility (*k* = 0.811). One study of heart failure patients showed no evidence of improved mean or hourly urine output in the group receiving albumin. The second studied patients hypoproteinemic with ARDS and demonstrated an improved fluid balance in 3 days, improved oxygenation status, and improved serum albumin level in patients treated with albumin. No significant differences were found for other outcomes. No studies evaluating colloids other than albumin were found.

**Conclusions:**

Our review is limited by the small number of high-quality RCTs available to study this clinical question, both of which only studied albumin. High-quality RCTs are required to evaluate the effect of albumin as well as other colloids as an adjunct to diuresis in a general ICU population.

**Electronic supplementary material:**

The online version of this article (doi:10.1186/2052-0492-2-37) contains supplementary material, which is available to authorized users.

## Background

Many critically ill patients require volume resuscitation with crystalloids, colloids, or blood products to treat the underlying condition which necessitated intensive care unit (ICU) admission. This aggressive resuscitation can lead to volume overload with marked peripheral edema and pulmonary edema and has been associated with the development of the acute respiratory distress syndrome (ARDS), as well as higher mortality compared to patients without evidence of volume overload [1, 2]. Observational data from a large European database suggests that positive fluid balance is among the most important prognostic variables for ICU mortality [3], and a retrospective review of the use of intravenous (IV) fluids during the first 4 days of sepsis care in the VASST study showed that a more positive fluid balance at both 12 h and day 4 correlated significantly with mortality [1]. Extending to the ARDS population, it is known that positive fluid balance in addition to a high tidal volume ventilatory strategy is associated with worse outcomes [2], and randomized controlled trial data from Wheeler et al. has shown a conservative IV fluid strategy to be of benefit with respect to improved lung function and duration of mechanical ventilation strategy in a broad ARDS population [4]. More recent data has suggested that in patients with acute lung injury complicating septic shock, adequate initial fluid resuscitation coupled with conservative late fluid management results in optimal outcomes [5]. Thus, in ICU patients without shock, maintenance of a euvolemic state and diuresis of excess fluid received during initial resuscitation is potentially of benefit, with loop diuretics such as furosemide being the standard therapy.

For such patients, a strategy of hyperoncotic colloid infusion followed by a diuretic infusion, such as furosemide, makes physiologic sense. Hyperoncotic colloid promotes redistribution of fluid from edematous peripheral tissues into the vascular compartment, where it can then be filtered at the glomerulus and excreted. This strategy has face validity for all edematous critically ill patients, but particularly so for those in whom critical illness and malnutrition have lead to hypoproteinemia. Low serum protein levels can result in lower vascular oncotic pressure and a tendency for fluid to shixft into the interstitial compartment [6].

Several colloids have the potential to increase colloid osmotic pressure (COP). Albumin has several potential advantages, as a naturally occurring protein whose levels tend to drop in the critically ill due to the effects of circulating inflammatory mediators [6]. Furthermore, furosemide itself is heavily protein-bound, and in hypoproteinemic patients, this results in an increased volume of distribution and lower concentrations of the diuretic in the loop of Henle [7]. The addition of albumin to furosemide has been shown to improve the volume of diuresis in several patient populations, including patients with renal failure [8–10] and cirrhosis [11]. Data from our institution shows that the administration of 100 mL of 25% albumin results in sustained increase in COP of 2.56 mmHg for up to 6 h [12]. Other colloids, notably synthetic hydroxyethyl starches also have the potential to improve COP. In addition to often being less expensive than albumin, they have been shown to have similar effects on COP in critically ill patients, increasing by 1.8 +/-3.1 mmHg for a duration of roughly 4 h [13]. No data about the effects of gelatins or human plasma upon COP could be found.

Thus, in critically ill patients, there is a rationale for studying the addition of colloids, including albumin, synthetic starches, gelatins, or plasma, to standard diuretic therapy in order to improve physiologic endpoints such as volume of diuresis, fluid balance, oxygenation status, as well as clinical outcomes such as ventilator-free days and mortality. However, observational studies have had mixed results [14–17]. Our goal in this systematic review is therefore to assess the highest quality current evidence in this regard, specifically assessing randomized controlled trials, which are at lower risk of bias compared to observational trials.

## Methods

### Eligibility criteria

We included trials with the following characteristics:

1. Type of studies: Randomized, controlled parallel group or crossover trials
2. Population: Adult patients in the ICU with the characteristics - not in shock (as defined by patients not on vasopressors, or on only low-dose vasopressors; without need for IV fluid bolus or blood product transfusion >24 h), without cirrhosis or the nephrotic syndrome
3. Intervention: Intravenous colloid therapy (human albumin, synthetic starches) plus diuretic therapy
4. Control: Diuretic alone or diuretic plus placebo
5. Outcomes: Prespecified outcomes included the following:
  - The effect on fluid balance, including urine output and weight loss during the treatment period, and any other measurements of volume status used by study authors; effects on hemodynamics, including the need for fluid replacement or blood product transfusion during or within 48 h of therapy, as well as hypotension, tachycardia, use of vasoactive agents; and effect on patient oxygenation (FiO_2_, PaO_2_/FiO_2_ ratio, oxygenation index).
  - Secondary outcomes: effects of serum protein, albumin, or colloid osmotic pressure as well as any chemistry data (serum urea, creatinine, electrolytes). Ventilator-free days, days in ICU, and ICU and total mortality data, if available were also collected.

### Search strategy-identification of studies

We conducted a search of MEDLINE (1946 to February 2013), Embase (1980 to February 2013), Cochrane Central Register of Controlled Trials as well as Google Scholar for all trials published from database inception to February 2013. Search strategies for each database can be found in Additional file 1: Appendix 1. Abstracts for meetings of American College of Chest Physicians (2003–2012), Society of Critical Care Medicine, American Thoracic Society (2009–2012), and Critical Care Canada Forum (2009–2012) were also hand-searched in duplication for relevant articles. The references of articles reviewed for eligibility were hand-searched in duplicate for further potentially relevant articles. Studies could be published in journals or in abstract form with no language restrictions. Studies of any methodological quality were considered eligible for review; however, only data from studies of moderate to low risk of bias were considered for pooling in a meta-analysis. No language restrictions were applied.

Obvious duplicates of retrieved studies were discarded. Study investigators, study time periods, population characteristics, and study methodology were closely examined to ensure that multiple reports of the same experimental data were not included. The retrieved studies were screened in duplication by the two reviewers (SO, IM) for relevance. Studies considered to be relevant were then assessed in duplication for eligibility. Provision was made for third-party review in the event of primary reviewer disagreement on the eligibility, but this was not necessary. Reviewers were not blinded as to article authors, journal, or results when screening studies for eligibility. Kappa statistics were calculated ensure inter-rater reliability of study relevance and study eligibility.

### Data extraction

Data were collected on standardized forms in duplicate by the reviewers. Lead authors of any studies that were missing essential data were contacted *via* their contact information given on the study.

### Methodologic quality assessment

Overall risk of bias of individual studies was assessed according to the tool used for the Cochrane Database of Systematic Reviews with regards to random sequence generation, allocation concealment, blinding of participants and personnel, incomplete outcome data, and selective reporting [18]. Studies were assessed independently by both reviewers and reported as being at a ‘high’, ‘low’, or ‘uncertain’ risk of bias. Disagreements between reviewers were settled by a third party.

### Statistical analysis

Data from eligible studies were entered into Revman Version 5.1, the software program used by the Cochrane Collaboration to perform systematic reviews [19]. We prespecified our statistical analysis for our primary and secondary outcomes. Study heterogeneity was to be assessed for each of the primary and secondary outcomes of interest and reported using I^2^ calculations, with values greater than 50% indicating substantial heterogeneity. For outcomes not found to have significant heterogeneity, summarized outcomes (standardized mean difference for continuous variables, relative risk for dichotomous variables) and 95% confidence intervals were to be calculated using a random-effects model. Prespecified subgroups for analysis included patients with hypoalbuminemia or hypoproteinemia and patients with ALI/ARDS, liver failure, or congestive heart failure, as they were considered the most likely to benefit from intravenous colloid.

## Results

### Search results

**Figure 1:**
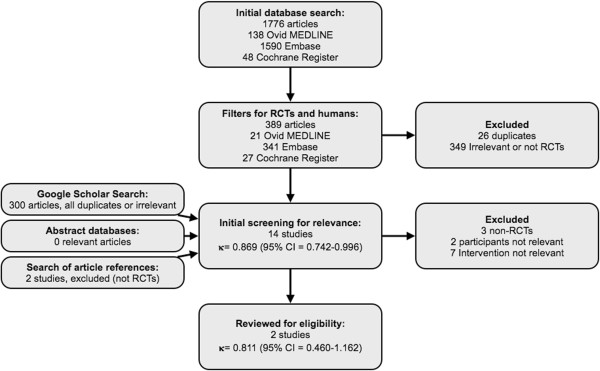
**Flowchart of studies selected for the systematic review.** RCT, randomized controlled trial.

The initial database search resulted in 1,755 articles, which narrowed to 363 once filters limiting results to clinical trials in humans were applied. The Google Scholar search revealed 300 articles, all of which were duplicates or not considered to be relevant for screening. Abstract databases revealed no further relevant publications. Hand searches of the article references revealed two further published abstracts considered to be relevant for screening. Initial screening by the reviewing authors resulted in 14 studies that were considered relevant for eligibility screening. Kappa statistic for agreement between the two authors was 0.869 (95% confidence interval (CI) = 0.742–0.996). After formal eligibility assessment by both reviewers, two studies were considered eligible for the systematic review. The Kappa statistic for agreement was 0.811 (95% CI = 0.460–1.162). There was complete agreement between the reviewers with regard to the overall risk of bias of the studies (Figure 1).

### Risk of bias assessment

**Figure 2:**
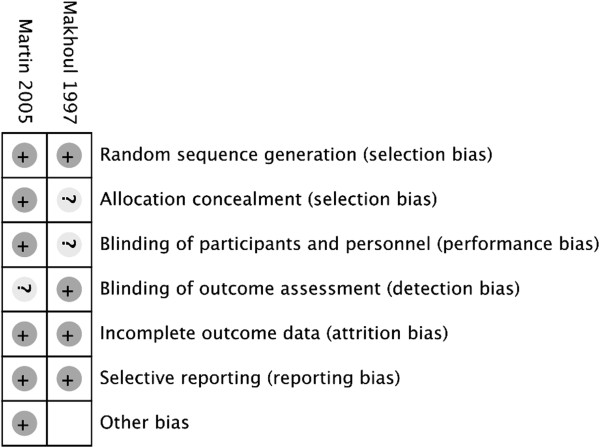
**Risk of bias assessment.**

The two RCTs were found to be at low to moderate risk of bias (Figure 2). Specific ratings of methodological assessment are listed in Table 1. Study authors were contacted to clarify any aspects of the studies that were ambiguous in the published manuscripts; however, further details were not available.

**Table 1:** **Study results**

Outcome	Albumin with diuretic	Diuretic alone	***p*** value	Source
Fluid balance & urine output				
Mean output/24 h	2,920 ± 1,172.1	3,672.5 ± 1,353.7	n.s.	[21]
Net balance at day 3	-5,480 mL	-1,490	<0.01	[20]
Hemodynamic measurements				
ΔCI at day 3	-0.1	0.2	n.s.	[20]
ΔMAP at day 3	-1.5	0.6	n.s.	[20]
Fluid boluses needed	11	35	Not reported	[20]
Oxygenation measurements				
ΔPaO_2_/FIO_2_ at 24 h	43	-24	<0.01	[20]
Serum albumin				
Increase in albumin at day 3	13 g/L	3 g/dL	<0.001	[20]
Colloid osmotic pressure				
Increase in COP at day 3	6.7	2.1	<0.01	[20]
Rate of furosemide infusion at day 5 (mg/h)	4.9	6.7	<0.005	[20]
Ventilator-free days				
30 day follow-up	5.5	1	n.s.	[20]
ICU mortality				
Total mortality in ICU	7 (35%)	9 (45%)	n.s.	[20]

CI, cardiac index; MAP, mean arterial pressure; PaO_2_/FIO_2_, partial pressure arterial oxygen/fraction of inspired oxygen ratio; COP, colloid osmotic pressure.

### Study results

The larger of the two studies (Martin [20]) randomized 40 patients with ARDS to 25 g of 25% albumin every 8 h or placebo. All patients received an infusion of furosemide (1 mg/mL) titrated to meet a net fluid loss of >1 kg per 24 h with a maximum dose of 8 mg/h [20]. The study by Makhoul et al. randomized 30 mechanically ventilated patients with congestive heart failure to one of three arms: one of intermittent furosemide; one of continuous furosemide infusion, and one of furosemide infusion plus IV albumin infusion. The regimen used was a bolus of 1 mg/kg, followed by an infusion of 0.1 mg/kg/h thereafter. Clinicians could increase the dose every 2 h PRN to keep urine output greater than 1 mL/kg/h. Patients randomized to continuous infusion plus albumin received furosemide at same infusion rate, but the furosemide was mixed into a solution of albumin, 12.5 g albumin per 250 mg of furosemide [21].

Study results are summarized in Table 1. Insufficient data was available in the study data (published or unpublished) to allow for a meta-analysis of any of the pre-specified outcomes. In the Makhoul study, there were no differences in mean total urine output at 24 h, although there was a trend towards a lower dose of furosemide in the group assigned to IV albumin infusion. There were no differences between groups with regard to electrolyte imbalances. In the study by Martin, albumin treatment resulted in increased serum albumin levels and colloid osmotic pressure at 72 h. There was a non-significant trend towards the reduction of the furosemide dose required by the group assigned to albumin (5.2 vs. 7.0 mg/h, *p* = 0.06). There were improvements in the net fluid balance at day 3 (-5.480 vs. -1.490 L, *p* < 0.01). There was also an improvement in the oxygenation at 24 h (change in P/F ratio 43 vs. -24, *p* < 0.01). There were no differences between the groups in cardiac index or mean arterial pressure though there were fewer fluid boluses needed in the group assigned to albumin treatment (11 vs. 35, *p* value not reported). There were no statistically significant differences between the groups with regard to ventilator-free days or ICU mortality.

## Discussion

The strengths of our review are its structured clinical question, thorough systematic search, and independent assessment of study relevance, eligibility, and quality with good agreement between reviewers. Our review is limited by the small number of high-quality RCTs available to study whether the addition of intravenous colloid to diuretic infusion, in comparison to diuretic infusion alone, is of benefit in ICU patients without shock. The two studies that do exist both assessed the effects of albumin, without comparison to other colloids. They were also small and limited to populations with either ARDS or congestive heart failure thus limiting generalizability to a broader critically ill population. Only one of the two high-quality RCTs suggests benefit for the use of albumin in addition to diuretics to improve the physiologic parameters of fluid balance, oxygenation, as well as possibly hemodynamic stability. The significance of this with regard to other patient important outcomes, such as ventilator-free days or mortality, is unknown. Thus, the routine administration of colloids in addition to diuretic cannot be recommended based on the above evidence, although the use of albumin may be considered in hypoproteinemic patients with ARDS who have a poor response to diuresis. Based on the lack of evidence, we are unable to comment on the use of other colloids, such as plasma or starches as an adjunct to diuresis in critically ill patients, although physiologic rationale for them are similar to those of albumin.

For a diuretic strategy that is used frequently, we were surprised at the paucity of high-quality RCT evidence. Further trials are needed to assess whether or not a strategy of albumin combined with furosemide results in improved patient-important outcomes and to determine if the potential benefit of albumin in addition to diuretic can be recommended to a broader population of critically ill patients. Such a trial should have a simple, practical protocol and include a wide range of critically ill patients, as volume overload is common in the ICU. Ideally, such a trial would also be powered to assess patient-important as well as physiologic outcomes.

For hydroxyethyl starches, smaller pilot studies to evaluate their effects upon COP and fluid balance are needed before proceeding to larger clinical trials. Given recent trials demonstrating potential harms of starches for resuscitation in critically ill patients; however, such trials are unlikely to be conducted [22, 23]. Based upon their physiologic effects upon COP, one could theoretically consider their use in patients for whom the use of blood products is unacceptable, though we would not recommend it at the present time.

Finally, for other colloids, such as gelatins or human plasma, no studies investigating their clinical or physiologic effects upon COP or diuresis in critically ill patients could be found. We are thus unable to recommend their use as an adjunct to diuresis in the critically ill population.

## Conclusions

Although there is good physiologic rationale for the use of colloids, particularly albumin, in addition to diuretics in critically ill patients with hypoproteinemia, there is a paucity of randomized controlled trials to support this practice. Two small trials comparing albumin to placebo suggest improvement of physiologic parameters, but they do not provide enough data for a meta-analysis. Further trials are needed to assess whether or not a strategy of albumin combined with furosemide results in improved patient-important outcomes and to determine if the potential benefit of albumin in addition to the diuretic can be generalized to a broader population of critically ill patients. No RCTs investigating the effects of starches, gelatins, or plasma as an adjunct to diuresis in the critically ill population exist, and small pilot studies assessing their physiologic effects upon COP and fluid balance are required before any recommendations to their use can be made. Given recent trials demonstrating the potential harms of hydroxethyl starches, such trials are unlikely, though they may still be considered for human plasma and gelatins.

## Electronic supplementary material

Additional file 1: Appendix 1: Search Strategy. (PDF 74 KB)

## Abbreviations

ARDS: acute respiratory distress syndrome
FiO_2_: fraction of inspired oxygen
ICU: intensive care unit
IV: intravenous
PaO_2_/FiO_2_ or P/F ratio: partial pressure of oxygen to fraction of inspired oxygen ratio.
