# Supplementary material for: Colloids to improve diuresis in critically ill patients: a systematic review
Source: J Intensive Care. 2014 Jun 10;2:37. doi: 10.1186/2052-0492-2-37 (PMC4424729; doi:10.1186/2052-0492-2-37)
Supplement: Supplementary file 1 — Additional file 1: Appendix 1: Search Strategy. (PDF 74 KB) [file 40560_2014_5044_MOESM1_ESM.pdf]

Appendix 1 - Search Strategy

Filters to include studies only including humans, as well as study type (eg. RCT) were applied after the search algorithm.

A Google Scholar search was conducted February 2, 2013 with a pre-specified independent review of the first 30 pages by both authors, as per the following search algorithm:
(critical care OR intensive care OR ICU OR septic OR sepsis) AND (furosemide OR lasix OR diuretic or diuresis) AND (colloid OR hydroxyethyl OR starch OR voluven OR pentaspan OR albumin) AND (RCT OR randomized OR TRIAL OR controlled)

OVID Medline (1946-January 2013)

1. critical care/ or intensive care/
2. Critical Illness/
3. exp Intensive Care Units/
4. exp Shock/
5. ICU.mp.
6. (critical* adj3 (care or ill*)).tw.
7. (intensive adj3 care).tw.
8. exp Sepsis/
9. acute lung injury/ or respiratory distress syndrome, adult/
10. exp multiple trauma/
11. postoperative/
12. or/1-11
13. Serum Album#n/
14. Hetastarch/
15. album#n.mp.
16. (hydroxyethyl* or colloid or starch or pentaspan or hetaspan or voluven).mp.
17. or/13-16
18. Furosemide/
19. exp Diuretics/
20. diuresis/ or urination/
21. (lasix or furosemide or f#mide or diur* or loop).mp.
22. or/18-21
23. 12 and 17 and 22

OVID EMBASE (1980 to 2013 Week 07)

1. exp intensive care/ or artificial ventilation/ or resuscitation/
2. exp critical illness/ or disease severity/
3. exp shock/ or cardiogenic shock/ or hemorrhagic shock/ or hypovolemic shock/ or septic shock/ or traumatic shock/
4. exp sepsis/ or systemic inflammatory response syndrome/ or bacteremia/ or fungemia/ or septic shock/ or septicemia/ or urosepsis/
5. exp intensive care unit/
6. (critical adj3 (care or illness)).tw.
7. (intensive adj3 care).tw.
8. sepsis.mp.
9. ICU.mp.
10. exp multiple trauma/
11. postoperative/
12. or/1-11

13. exp serum albumin/
14. exp hetastarch/
15. album#n.mp.
16. (pentaspan or voluven or hetaspan or hydroxyethyl or starch or colloid or pentastarch).mp.
17. or/13-16
18. exp diuresis/ or exp diuretic activity/ or exp diuretic agent/ or exp diuretic therapy/
19. (lasix or f#semide or diuretic or diuresis).tw.
20. or/18-19
21. 12 and 17 and 2-

OVID Cochrane (January 2013)

1. critical care/ or intensive care/
2. Critical Illness/
3. exp Intensive Care Units/
4. exp Shock/
5. ICU.mp.
6. (critical* adj3 (care).tw.
7. (critical* adj3 (ill*).tw
8. (intensive adj3 care).tw.
9. exp Sepsis/
10. acute lung injury/ or respiratory distress syndrome, adult/
11. exp multiple trauma/
12. postoperative.mp. or exp Postoperative Care/ or exp Postoperative Complications/ or exp Postoperative Period/
13. or/1-12
14. Serum Album#n/
15. Hetastarch/
16. album#n.mp.
17. (hydroxyethyl* or colloid or starch or pentaspan or hetaspan or voluven).mp.
18. or/14-17
19. Furosemide/
20. exp Diuretics/
21. diuresis/ or urination/
22. (lasix or furosemide or f#mide or diur* or loop).mp.
23. or/17-20
24. 13 and 18 and 23
